# Mitochondrial DNA transcription and translation: clinical syndromes

**DOI:** 10.1042/EBC20170103

**Published:** 2018-07-20

**Authors:** Veronika Boczonadi, Giulia Ricci, Rita Horvath

**Affiliations:** 1 Wellcome Centre for Mitochondrial Research, Institute of Genetic Medicine, Newcastle University, Central Parkway, Newcastle upon Tyne NE1 3BZ, U.K.; 2 Department of Clinical and Experimental Medicine, University of Pisa, Pisa, Italy

**Keywords:** mitochondrial translation, mitochondrial tRNA processing, mitochondrial tRNA modifications, mitochondrial tRNA synthetases

## Abstract

Diagnosing primary mitochondrial diseases is challenging in clinical practice. Although, defective oxidative phosphorylation (OXPHOS) is the common final pathway, it is unknown why different mtDNA or nuclear mutations result in largely heterogeneous and often tissue -specific clinical presentations. Mitochondrial tRNA (mt-tRNA) mutations are frequent causes of mitochondrial diseases both in children and adults. However numerous nuclear mutations involved in mitochondrial protein synthesis affecting ubiquitously expressed genes have been reported in association with very tissue specific clinical manifestations suggesting that there are so far unknown factors determining the tissue specificity in mitochondrial translation. Most of these gene defects result in histological abnormalities and multiple respiratory chain defects in the affected organs. The clinical phenotypes are usually early-onset, severe, and often fatal, implying the importance of mitochondrial translation from birth. However, some rare, reversible infantile mitochondrial diseases are caused by very specific defects of mitochondrial translation. An unbiased genetic approach (whole exome sequencing, RNA sequencing) combined with proteomics and functional studies revealed novel factors involved in mitochondrial translation which contribute to the clinical manifestation and recovery in these rare reversible mitochondrial conditions.

## Introduction

All eukaryotic cells contain both genomic and mtDNA and two separate protein synthesis machineries [1]. Mitochondria are essential eukaryotic organelles with the main function to produce the majority of cellular energy by oxidative phosphorylation (OXPHOS). While the majority of OXPHOS components (complexes I–IV), the ATP synthase (complex V), and various factors required for mtDNA maintenance (replication, transcription, copy number control) are encoded within the nucleus, 13 polypeptides, two ribosomal RNAs (mt-rRNAs), and 22 transfer RNAs (mt-tRNAs) are encoded within the mtDNA [1]. The expression of these molecules is fundamental for cellular functioning and is closely co-ordinated with nuclear gene expression. Mutations in some nuclear genes can cause secondary instability of the mitochondrial genome in the form of depletion (decreased number of mtDNA molecules in the cell), multiple deletions or accumulation of point mutations, which in turn leads to mitochondrial diseases inherited in a Mendelian fashion [2]. Expression of the mitochondrial genome is initiated by transcription of the mtDNA from bidirectional heavy and light strand promoters to produce two polycistronic transcripts [3]. Instead of initiating at individual gene-specific promoters, transcription of mammalian mtDNA initiates from single promoters for H- and L-strand transcription, and progresses around almost the entire length of the genome [4]. Following endonucleolytic processing individual mitochondrial mRNA (mt-mRNA), mitochondrial rNA (mt-rRNA), and mitochondrial tRNA (mt-tRNA) transcripts undergo post-transcriptional modifications [5,6]. The transcription machinery of the mtDNA is regulated by several transcription factors TFAM, TEFM and TFB2M and mitochondrial RNA polymerase POLRMT [7]. The 13 mtDNA encoded components of the OXPHOS machinery using the mitochondrial translation mechanism are synthesized within the mitochondria, with the participation of the mitoribosome [8,9]. The assembled mitoribosome translates the mt-mRNAs and synthesizes proteins that are rapidly inserted into the inner mitochondrial membrane and integrated into their relevant complexes to form the OXPHOS system [10].

**Figure 1:**
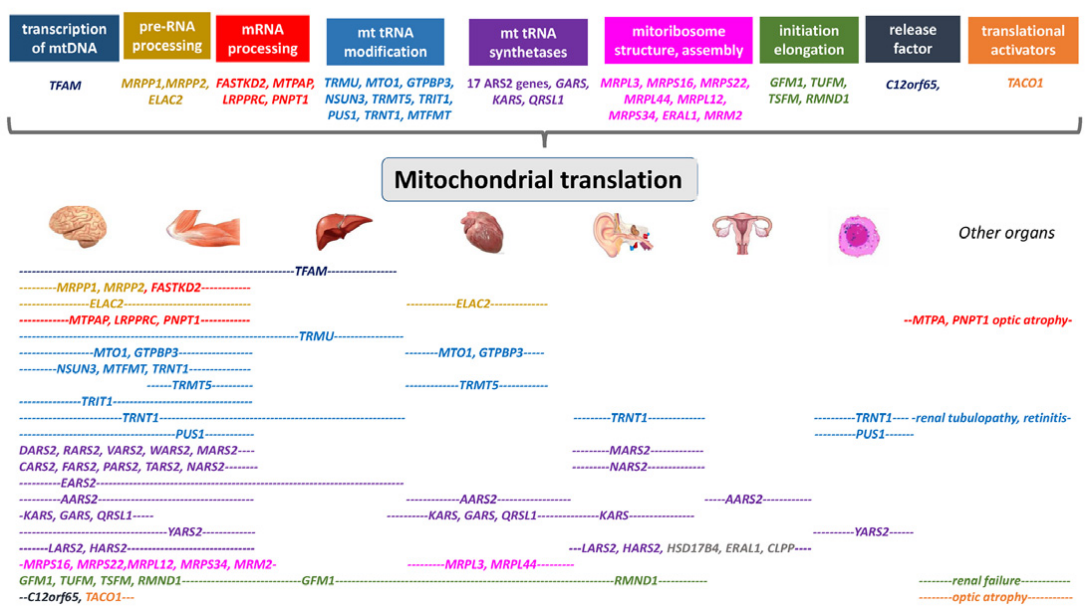
Summary of the genes and disease mechanisms implicated in mitochondrial translation deficiencies with associated clinical phenotypes

Approximately one-third of mitochondrial disorders have a presumed nuclear genetic defect of mitochondrial transcription and translation [11]. The identification of the molecular basis of this group has been particularly challenging and the recent availability of massively parallel sequencing have revealed several new disease genes, and unraveled new pathogenic mechanisms. Here, we present an overview of these tissue specific diseases (Figure 1).

## Defects of mitochondrial transcription

There are several genes involved in the initiation (*POLRMT, TFAM, TFB2B*) and elongation (*TEFM, MTERF1*) in transcription of mtDNA, however only mutations in *TFAM* have been shown to cause human diseases to date (Table 1) [12].

**Table 1:** Defects of mitochondrial transcription, pre-RNA, mRNA processing and stabilization

Gene	Protein	Clinical presentation	Age of onset	Mode of inheritance	OMIM	References
*TFAM*	Transcription factor A	Mitochondrial DNA depletion syndrome 15	Infancy	AR	617156	Stiles et al. (2016) [12]
*TRMT10C*	tRNA methyltransferase 10	Combined OXPHOS deficiency 30	Infancy	AR	616974	Metodiev et al. (2016) [13]
*HSD17B10 (MRPP2)*	NAD(P)(H)-dependent short-chain dehydrogenase/reductases	Global developmental delay, epilepsy, and cardiac involvement	Early childhood	AR	300256	Oerum et al. (2017) [14], Falk et al. (2016) [15]
*ELAC2*	RNase Z	Hypertrophic cardiomyopathy, hypotonia, lactic acidosis, delayed psychomotor development	Early childhood	AR	605367	Haack et al. (2013) [16], Shinwari et al. (2017) [17], Akawi et al. (2016) [18]
*FASTKD2*	fas activated serine-threonine kinase domain 2 protein	Later onset, milder MELAS (mitochondrial encephalomyopathy, lactic acidosis and stroke-like episode)-like syndrome with seizures, stroke-like episodes and optic atrophy. Mitochondrial encephalomyopathy with developmental delay, hemiplegia, convulsions, asymmetrical brain atrophy	Childhood	AR	612322	Ghezzi et al. (2008) [19], Yoo et al. 2017 [20]
*MTPAP*	Mitochondrial poly-A polymerase	Progressive spastic ataxia with optic atrophy	Juvenile or early childhood	AR	613672	Crosby et al. (2010) [21]
*LRPPRC*	Leucine-rich PPR-motif containing protein	Leigh syndrome French–Canadian variant (LSFC)	Infantile	AR	220111	Mootha et al. (2003) [22], Olahova et al. (2015) [23], Han et al. (2017) [24]

OMIM, Online Mendelian Inheritance in Man; AR, autosomal recessive.

### TFAM

The *TFAM* gene encodes a mitochondrial transcription factor essential for initiating mtDNA transcription, replication, and nucleoid packaging [25]. Pathogenic mutations in *TFAM* are linked to an autosomal recessive disorder with infantile-onset progressive fatal liver failure. Infants were born with intrauterine growth restriction and developed hepatopathy with elevated transaminases, conjugated hyperbilirubinemia, and hypoglycemia. Liver failure and death occurred in early infancy [12]. The mtDNA copy number has been shown to be decreased in patient liver, muscle, and fibroblasts. Liver biopsy shows cirrhosis, micro- and macrovesicular steatosis and cholestasis and abnormal mitochondrial morphology on electron microscopy. Biochemical enzymology in muscle showed increased citrate synthase activity and borderline reduced RC enzyme activities [12]. Based on these findings, it is likely, that mutations in other genes involved in mitochondrial transcription also result in low mtDNA copy numbers and a combined defect of the enzymes of the respiratory chain.

It has been implicated that TFAM-mediated alterations may be an important mechanism in neurodegeneration in Alzheimer, Huntington, Parkinson, and other neurodegenerative diseases [13]. Because altered TFAM and mtDNA levels have been detected in multiple models of neurodegeneration, we suggest that the regulation of TFAM may be a key mechanism in disease pathomechanism or progression [13].

## Maturation of the primary transcript: pre-RNA processing

Transcription of the mitochondrial genome generates large polycistronic transcripts punctuated by the 22 mt-tRNAs that are conventionally cleaved by the RNase P-complex and the RNase Z activity of ELAC2 at 5′ and 3′ ends, respectively (Table 1) [5,6].

### TRMT10C/MRPP1

Mutations in *TRMT10C* (encoding the mitochondrial RNase P protein 1 (MRPP1)) were reported in infants presenting at birth with lactic acidosis, hypotonia, feeding difficulties, and deafness [13]. Both individuals died at 5 months after respiratory failure. MRPP1, along with MRPP2 and MRPP3, form the mitochondrial ribonuclease P (mt-RNase P) complex that cleaves the 5′ ends of mt-tRNAs from polycistronic precursor transcripts. Analyses of fibroblasts from affected individuals harboring *TRMT10C* missense variants revealed decreased protein levels of MRPP1 and an increase in mt-RNA precursors indicative of impaired mt-RNA processing and defective mitochondrial protein synthesis [13].

### HSD17B10/MRPP2

MRPP2 (also known as HSD10/SDR5C1) belongs to the short-chain dehydrogenase/reductases (SDR) family and is involved in the catabolism of isoleucine and steroid metabolism [14]. MRPP2 also interacts in a complex with MRPP1 (TRMT10C) and MRPP3 (also known as PRORP), proteins involved in 5′-end processing of mitochondrial precursor tRNA [5].

A Caucasian boy with intractable epilepsy and global developmental delay carried a novel p.(Lys212Glu) mutation in the X-linked gene, *HSD17B10* encoding for mitochondrial SDR5C1 [15]. Mutations in *HSD17B10* lead to a metabolic disorder of fatty and amino acid metabolism, and affect an essential subunit of human mitochondrial RNase P, the enzyme responsible for 5′-processing and methylation of purine-9 of mt-tRNAs. The pathogenicity of the mutation is due to a general mitochondrial dysfunction caused by reduction in maturation of mt-tRNAs [15].

Two additional patients were reported with variable severity of developmental delay, epilepsy, and cardiac involvement. As a hallmark of the disease, urinary organic acid analysis showed elevated levels of 2-methyl-3-hydroxybutyric acid and tiglylglycine, and abnormalities were also detected in the acyl-carnitine spectrum in some cases [14].

### ELAC2 (RNase Z)

Mt-tRNAs are cleaved by the RNase Z activity of ELAC2 at their 3′ ends [17]. Mutations in *ELAC2* have been originally identified in five individuals with infantile hypertrophic cardiomyopathy and complex I deficiency and accumulation of mt-tRNA precursors in skeletal muscle and fibroblasts of the affected individuals, associated with impaired mitochondrial translation [17]. The association of severe, infantile cardiomyopathy and *ELAC2* mutations was supported by 16 additional cases, suggesting that it is a relatively frequent cause of severe infantile-onset hypertrophic or dilated cardiomyopathy. The p.(Phe154Leu) variant has a severe effect with poor prognosis [18]. Affected children in a consanguineous Pakistani family with a homozygous splice-site mutation in *ELAC2* presented with intellectual disability and minimal cardiac involvement [19].

## Maturation of the primary transcript: mRNA processing and stability

### FASTKD2

Mitochondrial encephalomyopathy with developmental delay, hemiplegia, convulsions, asymmetrical brain atrophy, and low cytochrome *c* oxidase (COX) activity in skeletal muscle were reported in patients with mutations in *FASTKD2*, encoding the fas activated serine-threonine kinase domain 2 protein [20]. FASTKD2 has a role in the assembly of the large ribosomal subunit and is required for 16S rRNA stability [26,27]. The tagged recombinant FASTKD2 protein co-localized with mitochondrial markers, and membrane potential-dependent mitochondrial import was demonstrated in isolated mitochondria *in vitro*. Later onset, milder mitochondrial encephalomyopathy, lactic acidosis and stroke-like episode (MELAS)-like syndrome with seizures, stroke-like episodes, and optic atrophy has been described in a Korean family with compound heterozygous mutations in *FASTKD2* [28]. FASTKD2 has been also implicated as a target for modulating neurodegeneration and memory loss in ageing and dementia [29]. Furthermore, FASTKD2 has been also shown to mediate apoptosis in breast and prostate cancers [21].

### MTPAP

In human mitochondria, polyadenylation of mRNA, undertaken by the nuclear-encoded mitochondrial poly(A) RNA polymerase, is essential for maintaining mitochondrial gene expression. An autosomal-recessive mutation has been identified in the *MTPAP* gene causing spastic ataxia with optic atrophy in the Old Order Amish population. Mt-mRNAs from affected individuals were shown to have severely truncated poly(A) tails [21]. Both mutated and wild-type MTPAP localized to the mitochondrial RNA-processing granules but the mutant protein generated only short oligo(A) extensions on RNA substrates, causing dysregulation of post-transcriptional expression leading to the reduction in respiratory chain complexes [30].

### LRPPRC

LRPPRC is a mt-mRNA chaperone that relaxes secondary structures [31] enabling polyadenylation and co-ordinated translation of mitochondrially encoded proteins [32,33]. In addition, *LRPPRC* has been documented in various tumors, contributing to the apoptosis resistance of human cancer cells [34] and it has been identified as an inhibitor of autophagy and mitophagy via interaction with the mitophagy initiator Parkin [35].

A homozygous founder mutation in the *LRPPRC* gene (c.1061C>T, p.(Ala354Val)) was identified as one of the first nuclear mitochondrial disease genes [22], associated with the French-Canadian variant of Leigh Syndrome (LSFC) and COX deficiency. LSFC is characterized by Leigh syndrome (a subacute neurodegeneration of the brainstem and basal ganglia), developmental delay, hypotonia, mild facial dysmorphism, and high mortality due to episodes of severe acidosis and coma that typically arise in the first year of life [22]. Subsequently, LSFC has also been described outside Quebec in ten patients from seven unrelated families of Caucasian, Pakistani, Indian, Turkish, and Iraqi origin [23] and in a Chinese boy with a milder phenotype [24]. The phenotype of these patients resembles LSFC, but in addition, neonatal cardiomyopathy or congenital malformations of the heart and the brain were reported. Decreased levels of mutant LRPPRC protein and impaired Complex IV enzyme activity were associated with abnormal COX assembly and reduced steady-state levels of numerous OXPHOS subunits in patients’ fibroblasts and skeletal muscle. In some patients complex I was also reduced, suggesting the role of *LRPPRC* in tissue-specific post-transcriptional regulation of mt-mRNAs [23].

## Diseases caused by abnormal tRNA modifications

Mt-tRNA modifications play a crucial role in regulating cellular energy delivery in response to local needs, and dysfunctional modifications may participate in the pathomechanism of mt-tRNA-related disorders (Table 2) [36].

**Table 2:** Defects of mt-tRNA modification

Gene	Protein	Clinical presentation	Age of onset	Mode of inheritance	OMIM	References
*TRMU*	tRNA 5-methylamino-methyl-2- thiouridy-late methyl-transferase	Reversible infantile liver failure	Infantile	AR	613070	Zeharia et al. (2009) [37] Schara et al. (2011) [38] Uusimaa et al. (2011) [39] Gaignard et al. (2013) [40]
*MTO1*	Mitochondrial translation optimization 1 homolog	Hypertrophic cardiomyopathy and lactic acidosis	Infantile	AR	614702	Ghezzi et al. (2012) [41], Baruffini et al. (2013) [42] O’Byrne et al. (2018) [43]
*GTPBP3*	GTP-binding protein 3	Hypertrophic or dilated cardiomyopathy, encephalopathy (hypotonia, developmental delay, seizures, visual impairment), lactate↑	Early childhood	AR	608536	Kopajtich et al. (2014) [44]
*NSUN3*	5-methylcytosine (m(5)C) methyltransferase	Developmental delay, microcephaly, failure to thrive, lactic acidosis, muscular weakness, external ophthalmoplegia, and nystagmus	Neonatal	AR	617491	van Haute et al. (2016) [45]
*TRMT5*	tRNA methyltransferase 5	Exercise intolerance, lactic acidosis, growth retardation, developmental delay, complex hereditary spastic paraparesis	Childhood neonatal	AR	611023	Powell et al. (2015) [46] Tarnopolsky et al. (2017) [47]
*TRIT1*	tRNA isopentenyl-transferase	Encephalopathy and myoclonic epilepsy, brain abnormalities	Childhood	AR		Yarham et al. (2014) [48] Kernohan et al. (2017) [49]
*TRNT1*	tRNA nucleotidyltransferase	Retinitis pigmentosa, erythrocitic microcytosis; sideroblastic anemia with B-cell immunodeficiency, periodic fevers, and developmental delay	Neonatal, juvenile	AR	612907	Chakraborty et al. (2014) [50] DeLuca et al. (2016) [51]
*PUS1*	Pseudouridine synthase	Myopathy, lactic acidosis, and sideroblastic anemia (MLASA1)	Early childhood to adult age	AR	608109	Bykhovskaya et al. (2004) [52] Fernandez-Vizarra et al. (2007) [53] Metodiev et al. (2015) [54]
*MTFMT*	Methionyl-tRNA formyltransferase	Leigh encephalopathy, white matter lesions, microcephaly, mental retardation, ataxia, and muscular hypotonia	Childhood	AR	611766	Tucker et al. (2011) [55] Neeve et al. (2013) [56] Haack et al. (2014) [57]

OMIM, Online Mendelian Inheritance in Man; AR, autosomal recessive.

### Wobble base modifications *(TRMU, MTO1, GTPBP3, NSUN3*)

#### TRMU

Reversible infantile liver failure is caused by autosomal recessive mutations in the tRNA 5-methylaminomethyl-2-thiouridylate methyltransferase [37–40] and the majority of these patients show complete spontaneous recovery if they survive the first year of life [58]. TRMU is an enzyme responsible for the thiouridylation of mt-tRNA^Glu^, mt-tRNA^Gln^, and mt-tRNA^Lys^, which requires cysteine. Cysteine is an essential amino acid in the first months of life, because of the physiologically low activity of the cystathionine γ-lyase (cystathionase) enzyme in infants [59]. The age-dependent, partially reversible clinical presentation of *TRMU* mutations resembles reversible infantile respiratory chain deficiency due to them. 14674T>C/G mutation in mt-tRNA^Glu^. Low dietary cysteine may be a common trigger of the clinical presentation of both diseases [60]. Mutations in *TRMU* have been also suggested to aggravate the deafness phenotype of the mitochondrial m.1555A>G 12S rRNA mutation [61], however the variants reported here were rather variants of unknown significance and had no involvement in liver disease.

#### MTO1

*MTO1* (mt-tRNA Translation Optimization 1), an evolutionarily conserved gene encodes the enzyme that catalyzes the 5-taurinomethylation of the wobble uridine base in mt-tRNA^Gln^, tRNA^Glu^, and tRNA^Lys^. This post-transcriptional modification increases the accuracy and efficiency of mtDNA translation [61].

The first patients carrying recessive mutations in the *MTO1* gene were identified in 2012 [41]. The clinical presentation was severe infantile hypertrophic cardiomyopathy. Two patients died within the first days of life, while the third unrelated subject showed marked improvement of the cardiomyopathy in childhood, and at the age of 19 years he suffered a stable hypertrophic cardiomyopathy with normal ejection fraction and moderate bilateral optic atrophy. Five additional patients were presented with hypertrophic cardiomyopathy and lactic acidosis in association with encephalopathy and psychomotor delay [42]. All patients complained of first symptoms soon after birth and two of them died in their first days of life. More recently, in a large cohort of 35 cases of *MTO1* deficiency [61], none of the patients had bi-allelic null variants suggesting that the complete loss of MTO1 is not viable. The most common features at presentation are lactic acidosis and hypertrophic cardiomyopathy with global developmental delay/intellectual disability (97%), feeding difficulties (49%), hypotonia (63%) failure to thrive (34%), seizures (34%), optic atrophy (52%), and ataxia (21%) and low activity of respiratory chain enzymes I, III, and IV. A subjective clinical improvement was observed in some patients on ketogenic diet and therapy with dichloroacetate [43].

#### GTPBP3

Mutations in *GTPBP3* are associated with a severe mitochondrial translation defect, due to the abnormal formation of 5-taurinomethyluridine (τm(5)U) in the anticodon wobble position of mt-tRNAs [44]. Eleven individuals from nine families were reported with recessive mutations in *GTPBP3*, encoding the mitochondrial GTP-binding protein 3 [44]. All patients presented with lactic acidosis and nine developed hypertrophic cardiomyopathy, but in contrast with individuals with mutations in *MTO1* (involved in the same modification), most individuals with *GTPBP3* mutations developed neurological symptoms and MRI involvement of thalamus, putamen, and brainstem resembling Leigh syndrome [44]. Affected individuals from eight out of nine families presented with combined respiratory chain complex deficiencies in skeletal muscle.

#### NSUN3

The recently characterized 5-methylcytosine (m(5)C) methyltransferase, NSun3 links m(5)C RNA modifications with energy metabolism [45]. Loss of function mutations in *NSUN3* a previously uncharacterized m(5)C methyltransferase, have been identified in a patient who developed combined developmental delay, microcephaly, failure to thrive, recurrent lactic acidosis, muscular weakness, external ophthalmoplegia, and nystagmus at 3 months of age with combined OXPHOS deficiency in skeletal muscle [45].

### Position 37 modifications *(TRMT5, TRIT1)*

#### TRMT5

Autosomal recessive mutations in the *TRMT5* gene (encoding tRNA methyltransferase 5) were reported in two patients with strikingly different clinical presentation [46]. While both affected individuals presented with lactic acidosis and evidence of multiple mitochondrial respiratory chain complex deficiencies in skeletal muscle, one presented with failure to thrive and hypertrophic cardiomyopathy in childhood, and the other was an adult with a life-long history of exercise intolerance. Recently, TRMT5 mutations were also linked to complex hereditary spastic paraparesis [47]. Mutations in *TRMT5* were associated with the hypomodification of a guanosine residue at position 37 (G37) of mt-tRNA, predominantly in skeletal muscle.

#### TRIT1

The first pathogenic mutation in *TRIT1* (encoding the tRNA isopentenyltransferase, responsible for i6A37 modification of some cytosolic and mt-tRNAs) has been identified in two siblings with encephalopathy and myoclonic epilepsy and severe combined mitochondrial respiratory chain defects [48]. It has been show that a previously reported pathogenic m.7480A>G mt-tRNA^Ser(UCN)^ mutation also acts by causing a loss of i6A37 modification, demonstrating that mt-tRNA^SerUCN^ is the substrate for TRIT1 [48]. Four individuals from three unrelated families ‘matched’ by GeneMatcher and MatchMakerExchange confirmed the role of *TRIT1* in human disease [49]. The patients had microcephaly, developmental delay, epilepsy, and decreased levels of selected mitochondrial proteins [49].

### CCA adding: *TRNT1*

TRNT1 (CCA-adding transfer RNA nucleotidyl transferase) enzyme deficiency is a complex metabolic disease caused by defective post-transcriptional modification of mitochondrial and cytosolic tRNAs [62]. Mutations in *TRNT1* cause congenital sideroblastic anemia, immunodeficiency, fevers, and developmental delay (SIFD) [50]. Further mutations in *TRNT1* have been reported in patients with a combination of abnormal blood cells (sideroblastic anemia, B lymphocyte or combined B and T immunodeficiency), metabolic crisis, and multisystem mitochondrial disease (retinitis pigmentosa, hepatosplenomegaly, exocrine pancreatic insufficiency, and renal tubulopathy [62,63–65]. Other clinical features include sensorineural deafness, cerebellar atrophy, brittle hair, partial villous atrophy, and nephrocalcinosis. *TRNT1* mutations cause a spectrum of symptoms ranging from a childhood-onset complex disease with manifestations in most organs to an adult-onset isolated retinitis pigmentosa presentation. Acute management of these patients includes transfusion for anemia, fluid and electrolyte replacement, immunoglobulin therapy, and potentially bone marrow transplantation. A defect of 3′-CCA addition to mt-tRNAs (tRNA(Cys), tRNA(LeuUUR) and tRNA(His)) demonstrates a novel pathomechanism [62].

### Pseudouridylation: *PUS1*

Pseudouridylate synthase 1 (PUS1) is an enzyme located in both nucleus and mitochondria, which converts uridine into pseudouridine in several cytosolic and mt-tRNA positions and increases the efficiency of protein synthesis in both compartments [66,52]. Myopathy, lactic acidosis, sideroblastic anemia (MLASA) syndrome is a rare autosomal recessive disease caused by recessive mutations in *PUS1* encoding the pseudouridine synthase 1 enzyme [52–54,66]. A similar phenotype has been observed in mutations in *YARS2* encoding the mitochondrial tyrosyl-tRNA synthetase [67]. Patients in consanguineous families of Persian, Jewish, and Italian origins presented with mental retardation, dysmorphic features, lactic acidosis, myopathy, sideroblastic anemia, and low activity of complexes 1 and 4 of the respiratory chain in muscles [53,54,68]. Some patients were reported with a mild phenotype of sideroblastic anemia and muscle weakness in adult age [54,69]. A double localization of PUS1 has been demonstrated, the isoform localized to the nucleus is predicted to be shorter (isoform 2) than the mitochondrial isoform, which contains an N-terminal mitochondrial targetting sequence. The structural differences in nuclear compared with mitochondrial isoforms of PUS1 may be implicated in the variability of the clinical presentations in MLASA [53].

### Formylation of the mitochondrial methionine tRNA (Met-tRNA^Met^)

The first mutations in the *MTFMT* gene in patients with Leigh syndrome and combined respiratory chain deficiency were reported by Tucker et al. [55]. In the past 5 years, several patients have been reported with *MTFMT* mutations and the clinical presentation is variable (Leigh encephalopathy, white matter lesions, microcephaly, mental retardation, ataxia, and muscular hypotonia) but often milder and later onset than other genetic forms of Leigh syndrome [56,57,70]. The mutations are usually loss-of-function mutations resulting in a severe decrease in MTFMT protein and reduced steady-state levels of complex I and IV subunits. The c.626C>T mutation has been detected in >80% of patients with MTFMT deficiency, and represents a relatively frequent cause of Leigh syndrome.

## Diseases of tRNA aminoacylation: mt-tRNA synthetases

Defects in nuclear genes encoding mitochondrial aminoacyl-tRNA synthetases (mt-ARSs) are increasingly linked to a variety of pediatric and adult onset tissue specific disorders [71]. Several recent reviews [72–75] presented detailed information, therefore here, we only provide a short summary of the most common phenotypes of mt-tRNA synthetase-related diseases (Table 3).

**Table 3:** Mutations in aminoacyl-tRNA synthetases

Gene	Protein	Clinical presentation	Age of onset	Mode of inheritance	OMIM	References
*DARS2*	Aspartyl-tRNA sythetase 2	- Leukoencephalopathy with brainstem and spinal cord involvement (LBSL) - Paroxysmal exercise-induced gait ataxia	Childhood or adulthood	AR	610956	Scheper et al. (2007) [76] Isohanni et al. (2010) [77] Miyake et al. (2011) [78] van Berge et al. (2014) [79] Shimojima et al. (2017) [80] Pinto et al. (2014) [81] Synofzik et al. (2011) [82]
*RARS2*	Arginyl-tRNA synthetase 2	Pontocerebellar hypoplasia type 6 (PCHD-6)	Neonatal or early childhood	AR	611523	Edvardson et al. (2007) [83] Rankin et al. (2010) [84] Cassandrini et al. (2013) [85] Li et al. (2015) [86] Lühl et al. (2016) [87]
*EARS2*	Glutamyl-tRNA synthetase 2	Leukoencephalopathy with thalamus and brainstem involvement and high lactate (LTBL); multiple congenital anomalies and multisystem dysfunction dysgenesis of corpus callosum	Congenital or infantile	AR	612799	Steenweg et al. (2012) [88] Talim et al. (2013) [89] Kevelam et al. (2016) [90] Güngör et al. (2016) [91] Şahin et al. (2016) [92]
*MARS2*	Methionyl-tRNA synthetase 2	Autosomal recessive spastic ataxia with leukoencephalopathy	Juvenile or adulthood	AR	609728	Bayat et al. (2012) [93] Webb et al. (2015) [94]
*FARS2*	Phenylalanyl-tRNA synthetase 2	Alpers syndrome, encephalopathy, epilepsy, lactic acidosis, spastic paraplegia	Neonatal or infantile	AR	611592	Elo et al. (2012) [95] Shamseldin et al. (2012) [96] Yang et al. (2016) [97]
*AARS2*	Alanyl-tRNA synthetase 2	- Hypertrophic cardiomyopathy - Ovario-leukodystrophy - Leukoencephalopathy with axonal spheroids and pigmented glia (ALSP)	Infantile to adulthood	AR	614096	Götz et al. (2011) [98] Taylor et al. (2014) [99] Dallabona et al. (2014) [100] Lynch et al. (2016) [101] Szpisjak et al. (2017) [102]
*YARS2*	Tyrosyl-tRNA synthetase	MLASA2, gastrointestinal difficulties, cardiomyopathy	Infantile	AR	613561	Riley et al. (2010) [67] Sasarman et al. (2012) [103] Shahni et al. (2013) [104] Riley et al. (2013) [105] Nakajima et al. (2014) [106]
*SARS*	Seryl-tRNA synthetase 2	- HUPRA syndrome (hyperuricemia, pulmonary hypertension, renal failure in infancy, and alkalosis) - Progressive spastic paresis	Infantile	AR	613845	Belostotsky et al. (2011) [107] Linnankivi et al. (2016) [108]
*HARS2*	Histidyl-tRNA synthetase 2	Perrault syndrome (sensorineural deafness, ovarian dysgenesis)	Juvenile or adulthood	AR	600783	Pierce et al. (2011) [109]
*LARS2*	Leucyl-tRNA synthetase	Perrault syndrome (sensorineural deafness, ovarian dysgenesis) hydrops, lactic acidosis, and sideroblastic anemia	Juvenile neonatal	AR	604544	Pierce et al. (2013) [110] Soldà et al. (2016) [111] Demain et al. (2017) [112] Riley et al. (2016) [113]
*TARS2*	Threonyl-tRNA synthetas	Mitochondrial encephalomyopathy Axial hypotonia and limb hypertonia, psychomotor delay, and high levels of blood lactate	Infantile	AR	612805	Diodato et al. (2014) [114]
*NARS2*	Asparginyl-tRNA synthetase	Non-syndromic deafness, Leigh syndrome, Alpers syndrome, infantile onset neurodegenerative disorder	Infantile	AR	612803	Sofou et al. (2015) [115] Vanlander et al. (2015) [116] Simon et al. (2015) [117] Mizuguchi et al. (2017) [118]
*CARS2*	Cysteinyl-tRNA synthetas	Combined oxidative phosphorylation deficiency-27 (COXPD27); severe epileptic encephalopathy and complex movement disorders	Juvenile	AR	612800	Coughlin et al. (2015) [119]
*IARS2*	Ileucyl-tRNA synthetase	- Skeletal dysplasia, infantile cataract, congenital neurotrophic keratitis, orbital myopathy, Leigh syndrome - CAGSSS syndrome	Adulthood or infantile	AR	616007 612801	Schwartzentruber et al. (2014) [120] Moosa et al. (2017) [121]
*VARS2*	Valyl-tRNa synthetase	Mitochondrial encephalomyopathy: psychomotor delay, epilepsy, mental retardation, growth hormone deficiency, hypogonadism	Juvenile	AR	612802	Diodato et al. (2014) [114] Baertling et al. (2017) [122] Alsemari et al. (2017) [123]
*WARS2*	Tryptophanyl-tRNA synthetase	- Autosomal recessive intellectual disability - Mitochondrial encephalopathy - Infantile-onset Parkinsonism	Infantile or juvenile	AR	604733	Musante et al. (2017) [124] Wortmann et al. (2017) [125] Theisen et al. (2017) [126] Burke et al. (2017) [127]
*PARS2*	Prolyl-tRNA synthetase	Non-syndromic hearing loss, Leigh syndrome, intellectual disability with epilepsy and severe myopathy, seizure	Infantile	AR	612036	Sofou et al. (2015) [128] Mizuguchi et al. (2017) [118]
*GARS*	Glycil-tRNA synthetase	- Charcot-Marie-Tooth disease, type 2D - Neuropathy, distal hereditary motor, type VA - Multisystem developmental delay, growth retardation- Lactic acidosis, cardiomyopathy, exercise intolerance	Adulthood, early childhood	AD AR	601472 600794	Antonellis et al. (2003) [129] Oprescu et al. (2017) [130] Nafisinia et al. (2017) [131] McMillan et al. (2014) [132]
*KARS*	Lysyl-tRNA synthetases	- Charcot-Marie-Tooth disease, recessive intermediate, B - Deafness, autosomal recessive 89 - Visual impairment and progressive microcephaly - Hypertrophic cardiomyopathy and combined mitochondrial respiratory chain defect	Adult, infantile, childhood	AR	613641 613916	Kohda et al. (2016) [133] Verrigini et al. (2017) [134] McMillan et al. (2015) [135] Santos-Cortez et al. (2013) [136] McLaughlin et al. (2010) [137]

OMIM, Online Mendelian Inheritance in Man; AR, autosomal recessive; AD, autosomal dominant.

Mutations in each of the 19 human mt-ARS genes have been reported in human disease [74]. Glycyl-(GARS) and lysyl tRNA (KARS) synthetase genes encode both cytosolic and mitochondrial ARS enzymes, suggesting links between protein syntheses in these two distinct cellular compartments. Other cytosolic ARSs are encoded by a set of genes distinct from those encoding mt-ARSs [138]. All mt-ARSs genes are located in the nucleus, synthesized in the cytosol, imported into the mitochondria by an N-terminal pre-sequence (mitochondrial targetting sequence, MTS), which is cleaved upon entry into the mitochondria [139].

Despite being ubiquitously expressed, mutations in these genes show an unexpected variety of phenotypes, including many neurological disorders affecting the white matter (*DARS2, EARS2, MARS2, AARS2*) or causing epileptic encephalopathy (*CARS2, FARS2, PARS2, TARS2, VARS2*), pontocerebellar hypoplasia (*RARS2*), or intellectual disability (*RARS2, WARS2*). While other characteristic phenotypes are sensori-neuronal hearing loss and ovarian failure (Perrault syndrome: *HARS2, LARS2*), mitochondrial myopathy, MLASA: *YARS2*, hyperuricemia, pulmonary hypertension, renal failure, alkalosis (HUPRA: *SARS2*), cardiomyopathy *(AARS2)*, or sensori-neural hearing loss *(MARS2, NARS2).* Besides the fact that new mutations are continuously discovered, neither the cause of the selective vulnerability, nor the exact molecular mechanisms leading to the diseases, are well understood. Degeneration of the central nervous system is speculated with early impairment of mitochondrial energy production that is crucial for myelination and maintenance of compact myelin [140]. Mutations in *DARS2* and *EARS2* result in very characteristic MRI phenotypes of leukoencephalopathy with brainstem and spinal cord involvement and lactate elevation (LBSL) [76] and leukoencephalopathy with thalamus and brainstem involvement and high lactate (LTBL) [141]. LBSL caused by mutations in *DARS2* is clinically characterized by slowly progressive pyramidal, cerebellar and dorsal column impairment, variably associated with delayed intellectual and/or motor development, cognitive impairment, epilepsy and peripheral neuropathy. The severity is ranging from early-onset severe disease, which can be fatal within the first years of life, to adult-onset forms [79,80]. The majority of patients carry a splice site mutation in intron 2, upstream of exon 3 [79]. Subgroups of patients with similar mutations (the common variants c.228-21_-20delTTinsC together with c.455G>T and c.492+2T>C) and a mild disease progression were identified. MRI abnormalities were correlated with the severity of the phenotype in mildly affected patients [81].

LTBL due to *EARS2* mutations is characterized by a biphasic clinical course [141,89,90]. Approximately one-third of patients suffered from hypotonia soon after birth, followed by spastic tetraparesis, dystonia, visual impairment, and seizures. The majority (two-third) of patients had normal or mildly delayed early development, disease-onset in the second half of the first year of life with clinical regression, spasticity, loss of milestones, sometimes seizures and irritability, and an improvement in symptoms and MRI abnormalities from the second year of life.

A founder mutation, p.(Arg590Trp) in *AARS2*, encoding the mt alanyl tRNA synthetase may predominantly affect the heart (infantile cardiomyopathy) [98,99], while other *AARS2* mutations are characterized by childhood- to adulthood-onset ataxia, spasticity, and dementia with frontal lobe dysfunction with leukoencephalopathy, cerebellar atrophy, and involvement of the corpus callosum on MRI [100]. Notably, all female patients also had ovarian failure. None of these cases suffered from a cardiomyopathy. Cardiomyopathy-associated mutations severely compromise aminoacylation, whereas partial activity is retained by the mutation combinations found in the leukodystrophy patients [142]. Similar molecular mechanisms may underlie the tissue specific manifestations of the other mt tRNA synthetases.

A few patients presented severe infantile multisystem disease predominantly affecting the heart and brain associated with combined OXPHOS enzyme deficiency have been reported recently with autosomal recessive mutations in the *QRSL1* gene [143,144]. No mitochondrial glutaminyl-tRNA synthetase (GlnRS) has been known and Gln-tRNA^Gln^ synthesis occurs via an indirect pathway involving QRSL1 (GatA). In this pathway, mt tRNA^Gln^ is first misaminoacylated by mt glutamyl-tRNA synthetase (GluRS) to form Glu-tRNA^Gln^, which is then followed by transamidation to Gln-tRNA^Gln^. This transamidation is processed by the hGatCAB heterotrimer. It has been shown that mutations in *QRSL1* (GatA), a component of hGatCAB were associated with severe transamidation activity defects [143].

### Perrault syndrome: *LARS2, HARS2 (HSD17B4, CLLP, ERAL1)*

Perrault syndrome is characterized by sensorineural hearing loss (SNHL) in males and females, and ovarian dysfunction in females. The SNHL is bilateral and ranges in severity from moderate with early-childhood onset to profound with congenital onset. Ovarian dysfunction ranges from gonadal dysgenesis (absent or streak gonads) manifesting as primary amenorrhea to primary ovarian insufficiency (POI) defined as cessation of menses before the age of 40. Fertility in affected males is reported as normal. Neurological features described in some affected women include developmental delay or intellectual disability, cerebellar ataxia, and motor and sensory peripheral neuropathy [145]. The diagnosis is confirmed by the presence of biallelic pathogenic variants in the genes *HARS2, HSD17B4, LARS2, ERAL1*, or *CLPP*. The fact that these seemingly different molecular mechanisms of mitochondrial translation can result in very similar, characteristic phenotypes raise the possibility of some common mechanisms.

## Mitoribosomal structure and assembly: *MRPL3, MRPS16, MRPS22, MRPL44, MRPL12, MRPS34, ERAL1*

Autosomal recessive mutations in nuclear encoded mitochondrial ribosomal proteins are rare and cause severe, infantile onset disease with growth retardation, neurological phenotypes (*MRPL3, MRPS16, MRPS22, MRPL12, MRPS34*) and cardiac involvement (*MRPL3, MRPL44*) (Table 4) [10]. Autosomal recessive mutations in the ribosomal assembly factor *ERAL1* have been associated with Perrault syndrome [145].

**Table 4:** Mutations in mitochondrial ribosomal proteins and ribosome assembly proteins

Gene	Protein	Clinical presentation	Age of onset	Mode of inheritance	OMIM	References
*MRPL*	Mitochondrial ribosomal protein L3	Hypertrophic cardiomyopathy and psychomotor retardation	Infantile	AR	614582	Galmiche et al. (2011) [146]
*MRPS16*	Mitochondrial ribosomal protein S16	Corpus callosum agenesia, hypothonia, and fatal neonatal lactic acidosis	Neonatal	AR	610498	Miller et al. (2004) [147]
*MRPS22*	Mitochondrial ribosomal protein S22	Cornelia de Lange-like syndrome Edema, cardiomyopathy and tubulopathy	Neonatal	AR	611719	Saada et al. (2007) [148] Smits et al. (2011) [149]
*MRPL44*	Mitochondrial ribosomal protein L44	Hypertrophic cardiomyopathy	Neonatal	AR	611849	Carroll et al. (2013) [150] Distelmaier et al. (2015) [151]
*MRPL12*	Mitochondrial ribosomal protein L12	Growth retardation and neurological deterioration	Neonatal	AR	602375	Serre et al. (2013) [152]
*MRPS34*	Mitoribosomal ribosomal protein S34	Leigh syndrome and combined OXPHOS defects	Neonatal	AR	611994	Richman et al. (2015) [153] Lake et al. (2017) [154]
*ERAL1*	mt-rRNA chaperone	Perrault syndrome	Childhood or adult	AR	607435	Newman et al. (2014) [143]

OMIM, Online Mendelian Inheritance in Man; AR, autosomal recessive.

## Translation initiation and elongation factors: *GFM1, TUFM, TSFM, RMND1*

The diseases caused by mutations in these factors are severe neonatal or infantile onset rare diseases affecting the brain (*GFM1, TUFM, TSFM, RMND1*), liver (*GFM1*), heart (*TSFM*), and other organs (*RMND1*) (Table 5) [11]. There are no diseases linked to mutations in translation termination factors to date. The most frequent gene defect in this group is caused by mutations in *RMND1* leading to a severe defect of mitochondrial translation in all tissues. The *RMND1* gene encodes an integral inner membrane mitochondrial protein that assembles into a large 240-kDa complex to support translation of the 13 polypeptides encoded on mtDNA [155,156]. Clinical and genetic features of 32 RMND1 patients from 21 pedigrees are hypotonia and developmental delay (75%), sensori-neural hearing loss (72%), nephropathy (53%), failure to thrive (53%), seizures (44%), microcephaly (41%), and spasticity (19%) [157]. The disease usually starts early, before 2 years of life, but patients with renal involvement show a later onset, better prognosis, and longer survival [157]. Four patients were successfully treated with kidney transplantation with a good clinical response.

**Table 5:** Mitochondrial translation initiation, elongation, termination, and release factors and translational activators

Gene	Protein	Clinical presentation	Age of onset	Mode of inheritance	OMIM	References
*GFM1*	Elongation factor G 1, mitochondrial (EFG1_mt_)	Encephalopathy with or without liver involvement	Neonatal	AR	609060	Coenen et al. (2004) [158] Valente et al. (2007) [159] Smits et al. (2011) [160]
*TUFM*	Elongation factor Tu, mitochondrial (EF-TU_mt_)	Lactic acidosis, leukoencephalopathy, and polymicrogyria	Neonatal	AR	610678	Valente et al. (2007) [159] Kohda et al. (2016) [161]
*TSFM*	Elongation factor Ts, mitochondrial (EF-Ts_mt_)	Encephalomyopathy, hypertrophic cardiomyopathy	Neonatal or childhood	AR	610505	Smeitink et al. (2006) [162] Smits et al. (2011) [160] Shamseldin et al. (2012) [163] Ahola et al. (2014) [164]
*RMND1*	Regulator of microtubule dynamics 1	Deafness, myopathy, renal involvement, cardiomyopathy and a severe biochemical defect Combined oxidative phosphorylation deficiency -11	neonatal	AR	614917 614922	Janer et al. (2012) [144] Garcia-Diaz et al. (2012) [145] Taylor et al. (2014) [99] Janer et al. (2015) [165] Gupta et al. (2016) [166] Ravn et al. (2016) [159] Vinu et al. (2018) [167]
*C12orf65*	Chromosome 12 ORF 65	Leigh syndrome, optic atrophy, ophthalmoplegia Spastic paraplegia with optic atrophy and axonal neuropathy (SPG55)	Infantile	AR	613559	Antonicka et al. (2010) [156] Pyle et al. (2014) [168] Shimazaki et al. (2012) [157] Spiegel et al. (2014) [169]
*TACO1*	Translational activator of COX1	Leigh syndrome	Juvenile	AR	612958	Weraarpachai et al. (2009) [170] Makrythanasis et al.(2014) [171]

OMIM, Online Mendelian Inheritance in Man; AR, autosomal recessive.

## Release factors: *C12orf65*

The *C12orf65* gene encodes a protein that is critical for the release of newly synthesized proteins from mitochondrial ribosomes and its deficiency was reported in patients with Leigh syndrome and optic atrophy [172], in autosomal recessive hereditary spastic paraplegia 55 (SPG55) [168] or Charcot-Marie-Tooth disease type 6 [169], or Behr’s syndrome (optic atrophy, spastic paraparesis, motor neuropathy, ataxia, ophthalmoparesis) [170]. The spectrum of *C12orf65*-related phenotypes includes the triad of early-onset optic atrophy, axonal neuropathy, and spastic paraparesis as key clinical features [170,173].

## Translational activators: *TACO1*

As mammalian mt-mRNAs do not have significant 5′ UTRs, alternate mechanisms exist to promote their translation. A defect in the translational activator of the mtDNA-encoded COX I subunit has been identified in a pedigree segregating late-onset Leigh syndrome and cytochrome *c* oxidase (COX) deficiency [174]. A single homozygous one-base-pair insertion has been identified in one large consanguineous Turkish family with teenage onset Leigh syndrome, cognitive decline, dystonia, and optic atrophy in *TACO1* for translational activator of COX I [174,175]. No other mutations have been reported to date worldwide to confirm the phenotype. However, our group has detected the previously described *TACO1* mutation in an additional consanguineous Turkish family (unpublished). The clinical phenotype in patients has been supported by the Taco1 mutant mice, which develop a late-onset visual impairment, motor dysfunction, and cardiac hypertrophy [176].

Mutation in PNPT1, which encodes a polyribonucleotide nucleotidyltransferase, impairs RNA import into mitochondria and causes respiratory-chain deficiency.

## Other mechanisms affecting mitochondrial translation

### Abnormal import of RNA into the mitochondria: *PNPT1*

*PNPT1* encodes the mitochondrial polynucleotide phosphorylase (PNPase), which is predominantly localized in the mitochondrial intermembrane space and is a 3′–5′ exoribonuclease which acts together with SUV3 to form the RNA degradosome within the mitochondrial matrix [177]. Two siblings with severe encephalomyopathy, choreoathetotic movements, and combined respiratory-chain defects carried a homozygous *PNPT1* missense mutation (c.1160A>G), which disrupts the trimerization of the protein. A defect of mitochondrial translation has been detected in the patient’s fibroblasts. Recently additional patients have been reported with recessive *PNPT1* mutations and the clinical presentation of early onset of severe axonal neuropathy, optic atrophy, intellectual disability, auditory neuropathy, and chronic respiratory and gut disturbances [178], and severe Leigh syndrome [179]. Specific RNA processing intermediates derived from mitochondrial transcripts of the ND6 subunit of Complex I, as well as small mRNA fragments, accumulated in the subject’s myoblasts indicates that PNPase activity is essential for the correct maturation of the ND6 transcript [179].

### Modification of rRNAs: *MRM2*

A homozygous missense mutation (c.567G>A; p.Gly189Arg) has been identified in a 7-year-old Italian boy with the clinical presentation of childhood-onset rapidly progressive encephalomyopathy and stroke-like episodes with multiple OXPHOS deficiency in skeletal muscle. *MRM2* encodes an enzyme responsible for 2′-O-methyl modification at position U1369 in the human mitochondrial 16S rRNA. Although a confirmation of the clinical phenotype in a second independent patient is still lacking, it is possible that mutations in *MRM2* cause a MELAS-like phenotype, and suggests the genetic screening of *MRM2* in patients with a m.3243 A > G negative MELAS-like disease [180].

## Summary

- Here we have illustrated the large variety of clinical presentations caused by defects of mitochondrial translation. More detailed understanding of the molecular mechanisms involved in mitochondrial translation may reveal some insights on the tissue specific phenotypes. Processing and modifications of mt-tRNAs may provide novel approaches to develop treatment to defects of mitochondrial translation.
- It has been recently shown that supplementation with cysteine (l-cysteine and N-acetyl-cysteine) improves mitochondrial translation in patients with reversible mitochondrial disease (*TRMU*, mt-tRNA^Glu^) and with m.3243A>G and m.8344A>G frequent mt-tRNA mutations [181], as absence of post-transcriptional modifications at the wobble positions of mt-tRNAs for Leu^UUR^ and Lys has been related to MELAS and myoclonic epilepsy with ragged-red fiber (MERRF), respectively.
- As another novel approach, leucyl tRNA synthetase is able to partially rescue defects caused by mutations in non-cognate mt-tRNAs and furthermore, a C-terminal peptide alone can enter mitochondria and interact with the same spectrum of mt-tRNAs as the entire synthetase in intact cells [182,183]. These data support the possibility that a small peptide may correct the biochemical defect associated with many mt-tRNA mutations, inferring a novel therapy for these disorders.

## Abbreviations

COX: cytochrome *c* oxidase
LBSL: leukoencephalopathy with brainstem and spinal cord involvement and lactate elevation
LSFC: French-Canadian variant of Leigh syndrome
LTBL: leukoencephalopathy with thalamus and brainstem involvement and high lactate
MLASA: myopathy, lactic acidosis, and sideroblastic anemia
MELAS: mitochondrial encephalomyopathy, lactic acidosis and stroke-like episode
mt-ARS: mitochondrial aminoacyl-tRNA synthetase
mt-mRNA: mitochondrial mRNA
mt-rRNA: mitochondrial rRNA
mt-tRNA: mitochondrial tRNA
OXPHOS: oxidative phosphorylation
PNPase: polynucleotide phosphorylase
SDR: short-chain dehydrogenase/reductase
